# Supplementary material for: Causal Relationship between Aging and Anorexia Nervosa: A White-Matter-Microstructure-Mediated Mendelian Randomization Analysis
Source: Biomedicines. 2024 Aug 16;12(8):1874. doi: 10.3390/biomedicines12081874 (PMC11351342; doi:10.3390/biomedicines12081874)
Supplement: Supplementary file 1 [file biomedicines-12-01874-s001.zip › Supplementary Figure/Figure S4/Singe SNP plot of MR analysis for TL to ubm-b-2092.pdf]

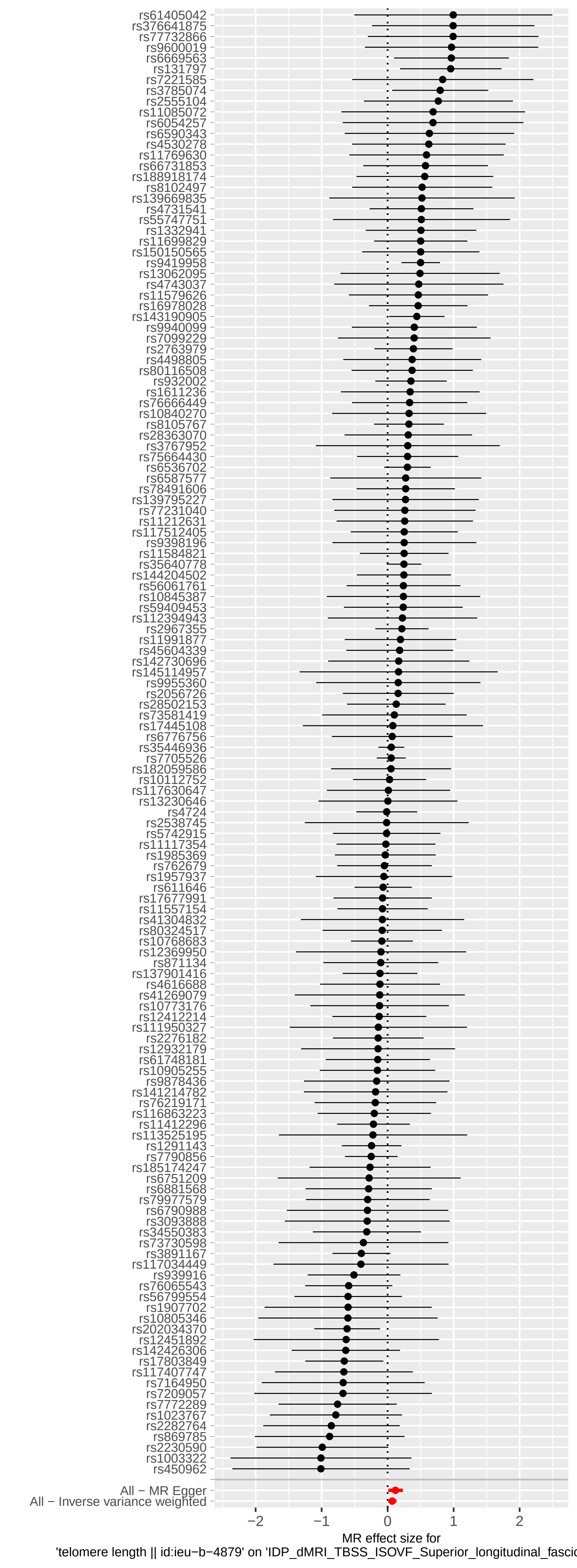
rs61405042
rs376641875
rs77732866
rs9600019
rs6669563
rs131797
rs7221585
rs3785074
rs2555104
rs11085072
rs6054257
rs6590343
rs4530278
rs11769630
rs66731853
rs188918174
rs8102497
rs139669835
rs4731541
rs55747751
rs1332941
rs11699829
rs150150565
rs9419958
rs13062095
rs4743037
rs11579626
rs16978028
rs143190905
rs9940099
rs7099229
rs2763979
rs4498805
rs80116508
rs932002
rs1611236
rs76666449
rs10840270
rs8105767
rs28363070
rs3767952
rs75664430
rs6536702
rs6587577
rs78491606
rs139795227
rs77231040
rs11212631
rs117512405
rs9398196
rs11584821
rs35640778
rs144204502
rs56061761
rs10845387
rs59409453
rs112394943
rs2967355
rs11991877
rs45604339
rs142730696
rs145114957
rs9955360
rs2056726
rs28502153
rs73581419
rs17445108
rs6776756
rs35446936
rs7705526
rs182059586
rs10112752
rs117630647
rs13230646
rs4724
rs2538745
rs5742915
rs11117354
rs1985369
rs762679
rs1957937
rs611646
rs17677991
rs11557154
rs41304832
rs80324517
rs10768683
rs12369950
rs871134
rs137901416
rs4616688
rs41269079
rs10773176
rs12412214
rs111950327
rs2276182
rs12932179
rs61748181
rs10905255
rs9878436
rs141214782
rs76219171
rs116863223
rs11412296
rs113525195
rs1291143
rs7790856
rs185174247
rs6751209
rs6881568
rs79977579
rs6790988
rs3093888
rs34550383
rs73730598
rs3891167
rs117034449
rs939916
rs76065543
rs56799554
rs1907702
rs10805346
rs202034370
rs12451892
rs142426306
rs17803849
rs117407747
rs7164950
rs7209057
rs7772289
rs1023767
rs2282764
rs869785
rs2230590
rs1003322
rs450962
All − MR Egger
All − Inverse variance weighted
−2
−1
0
1
2
MR effect size for
'telomere length || id:ieu−b−4879' on 'IDP_dMRI_TBSS_ISOVF_Superior_longitudinal_fascic
